# 2023 EULAR Recommendations for the Treatment of PsA: Advances and Pending Issues

**DOI:** 10.31138/mjr.050424.ert

**Published:** 2024-04-15

**Authors:** Philipp Sewerin, Xenofon Baraliakos

**Affiliations:** Ruhr-University Bochum, Bochum, Rheumazentrum Ruhrgebiet, Herne, Germany

**Keywords:** EULAR, recommendations, psoriatic arthritis

The “EULAR recommendations for the management of psoriatic arthritis with pharmacological therapies: 2023 update” were published in March 2024.^1^ The update was necessary due to new treatments and therapy strategies as well as new safety signals of the compounds included in the previous and the updated version of the recommendations. A total of 7 overarching therapeutic principles and 11 recommendations were developed, with some recommendations remaining unchanged, others being revised and new ones added. As a result, some of the long open questions with respect to a targeted treatment of patients with psoriatic arthritis (PsA) could be answered, although some points still remain controversial and other questions remained unanswered.

Firstly, the role of non-steroidal anti-inflammatory drugs (NSAID) was reclassified. NSAIDs should only be used as monotherapy for mild PsA and only for a short time period. This change to the previous guideline emphasises that the early use of disease-modifying therapy should not be delayed by prolonged use of NSAIDs. A maximum duration of only 4 weeks is now recommended for NSAID therapy and only in mild cases, after which disease-modifying antirheumatic drug (DMARD) therapy should be started at the latest. This change seems to be a logical consequence, particularly considering the numerous studies that also see a window of opportunity in PsA.^2^

In addition, somewhat surprisingly for some, the use of oral glucocorticoids is no longer recommended at all. On the one hand, this is understandable, as PsA patients have comparatively many comorbidities that can certainly be negatively influenced by glucocorticoids. In addition, with the exception of the polyarticular type of the disease, the effect is very limited and can lead to numerous unwanted side effects, especially in long-term therapy. Nevertheless, in many countries, glucocorticoids are still an integral part of the initial therapy for short-term use, provided that the glucocorticoids are quickly reduced and completely discontinued. Overall, the recommendation is long awaited, but may pose challenges for rheumatologists in daily clinical practice, especially in patients with highly active disease.

After initiation of conventional synthetic (cs)DMARD therapy and an inadequate response to it, therapy should be escalated to a biologic (b)DMARD with no further delay, with no preference for a particular mode of action, as there is a lack of clear evidence in favour of the preferred use of a particular treatment. To date, with a few exceptions, it is still unclear whether a tumour necrosis factor inhibitor (TNFi), Interleukin (IL)17 inhibitor (IL17i) or IL12/23i or IL23i should be started after Methotrexate (MTX) has been administered, which is why the updated guideline does not recommend a specific ranking of treatments in daily clinical practice in general. However, new clinical studies indicate preferences for specific clinical manifestations. For example, if there is severe skin involvement, IL12/23i or IL23i or IL17i are rather recommended. This opinion is based on various study programs that have shown an advantage of these therapy concepts in direct comparison with TNFi in cases of severe skin involvement. On the other hand, therapy with TNFi should be favoured in cases of concomitant severe uveitis or inflammatory bowel disease. This specification of the recommended bDMARD therapy is new and certainly a first step in the direction of a more personalised therapy.

Independently of this, the role of Janus kinase inhibitors (JAKi) has been reclassified. Based on the oral surveillance study,^3^ both the U.S. Food and Drug Administration (FDA) and the European Medicines Agency (EMA) issued numerous warnings and, in the course of the study, amended the summary of product characteristics (SmPC). This was based on increased rates of cardiovascular events and malignancies with tofacitinib compared with TNFi in a large international study in high-risk RA patients, although the results have not been reproduced in many real-world studies and are not yet available for selective JAKi. In addition, there are two safety analyses, one for upadacitinib and one for tofacitinib specifically for PsA, which do not currently show any specific safety signals.^4,5^ This is also true of the real-world studies published to date. No safety signals have been identified for either RA or PsA outside the ORAL Surveillance study. This applies to both cardiovascular events and malignancies.^6,7^ Nevertheless, the role of JAKi in PsA had to be re-evaluated. Given the availability of other treatment options and the safety data on JAKi, the guideline now recommends its use in peripheral disease only after failure of bDMARD therapy, taking into account the safety signals to date. However, the recommendations are slightly different for patients with primary axial PsA, where JAKi are recommended at almost the same level as bDMARDs, considering the safety signals. The authors suggest several reasons for this. Firstly, fewer MOAs have been shown to be effective in axial disease. Secondly, the authors assume that there are fewer comorbidities in primary axial PsA than in peripheral disease, as patients’ comorbidities are more similar to those with axSpA. The overall more cautious approach to categorising JAKi is understandable, but from our perspective it may certainly change again with subsequent guidelines and in particular when new safety data are available for the other JAKi.

Finally, the importance of therapy tapering in patients in clinical remission was also addressed in the current treatment recommendations. This topic is present in many recommendations and is being discussed controversial. For example, it has been shown that discontinuation of therapy in patients with persistent disease remission leads to a flare more frequently than continuation or prolonging therapy intervals.

Today, there are experts who do not recommend discontinuing immunomodulatory therapy for PsA for a number of reasons. The main arguments for this are the chronic nature of the disease with no current options of ‘cure’, as well as the numerous comorbidities that are negatively influenced by the disease activity in case of a flare. In addition, not all patients with PsA can be well controlled again with their previous therapy after a relapse. On the other hand, modern therapies undoubtedly pose major challenges for payers, so that discontinuation of therapy must be discussed if clinically possible. Irrespective of the costs, many patients in remission also request to discontinue a therapy that is not absolutely necessary. Nevertheless, we believe that the wording of the recommendations is appropriate, as it clearly focusses on the current evidence but does not make a definite recommendation due to the lack of conclusive evidence.

At the end of each guideline, numerous questions obviously remain unanswered, which are summarised in the research agenda. Overall, there is still a lack of evidence to truly recommend a personalised management. For example, the question of the definition of early PsA is explicitly raised. When do we really need to start treatment? Are individual patients with enthesitis and defined psoriasis already considered as having an early phase of PsA? How do we want to treat these patients? What role do imaging techniques play in this?

We are also recognising increasingly clear differences between the sexes. Women respond differently to certain therapeutic modalities at different times than men. In addition, different domains are affected to different degrees by women and men. There is a lack of prospective study data to answer this question. Do we have to treat women differently than men? Always or only in specific situations? What options do we have when it comes to questions about pregnancy and breastfeeding?

Then there is the question of the role of comorbidities. We now understand better that these are also very common in PsA compared to other diseases. We also understand that adequate treatment of the underlying disease can minimise the risk of progression of comorbidities. However, it remains unclear whether and which therapy we should select for which comorbidity. Prospective data is also lacking here.

In summary, the new EULAR recommendations summarise the existing evidence at the highest level and provide helpful guidance for daily clinical practice. Despite these important improvements, some points remain on the research agenda that cannot be answered yet. Numerous studies are already underway that will help us develop even clearer and more personalised treatment strategies in the next recommendations. The current ones are a big step in this direction.

## ABBREVIATIONS

b: biologic
cs: conventional synthetic
DMARD: Disease Modifying Antirheumatic Drugs
EMA: European Medicines Agency
FDA: U.S. Food and Drug Administration
i: Inhibitor
IL: Interleukin
MOA: Mode of action
JAKi: Janus Kinase Inhibitors
NSAID: Non-steroidal Anti-inflammatory Drugs
PsA: Psoriatic Arthritis
SmPC: Summary of Product Characteristics
